# 5-(4-Chloro­anilinomethyl­ene)-2,2-dimethyl-1,3-dioxane-4,6-dione

**DOI:** 10.1107/S1600536809023897

**Published:** 2009-06-27

**Authors:** Jin-Cheng Yang, Jian-You Shi, You-Fu Luo, Neng Qiu, Li-Juan Chen

**Affiliations:** a Department of Medicinal Chemistry, West China School of Pharmacy, Sichuan University, Chengdu 610041, People’s Republic of China; b State Key Laboratory of Biotherapy, West China Hospital, Sichuan University, Chengdu 610041, People’s Republic of China

## Abstract

The title compound, C_13_H_12_ClNO_4_, is approximately planar, with a dihedral angle of 8.23 (4)° between the mean plane of the amino­methyl­ene unit and the planar part of the dioxane ring. The dioxane ring has a half-boat conformation, in which the C atom between the dioxane O atoms is −0.464 (8) Å out of the plane of the other five atoms. In the mol­ecule there is an intra­molecular N—H⋯O hydrogen bond, involving the NH H atom and the adjacent dioxane carbonyl O atom. In the crystal, weak intermolecular C—H⋯O hydrogen-bonding contacts, result in the formation of sheets parallel to the *ab* plane.

## Related literature

For the synthesis of related compounds, see: Cassis *et al.* (1985 ▶). For the synthesis of related anti­tumor precursors, see: Ruchelman *et al.* (2003 ▶). For details of the formation of quinolin-4-ol derivatives by thermal cracking, see: De *et al.* (1998 ▶). For the structure of 5-(amino­methyl­ene)-2,2-dimethyl-1,3-dioxane-4,6-dione, see: da Silva *et al.* (2006 ▶).
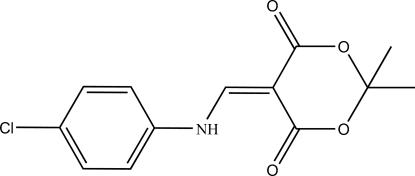


## Experimental

### 

#### Crystal data

C_13_H_12_ClNO_4_
*M* *_r_* = 281.69
Monoclinic, 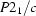

*a* = 13.439 (4) Å
*b* = 13.076 (3) Å
*c* = 7.723 (3) Å
β = 106.40 (2)°
*V* = 1302.0 (7) Å^3^
*Z* = 4
Mo *K*α radiation
μ = 0.30 mm^−1^
*T* = 293 K
0.46 × 0.44 × 0.22 mm

#### Data collection

Enraf–Nonius CAD-4 diffractometer
Absorption correction: spherical (*WinGX*; Farrugia, 1999 ▶)*T*_min_ = 0.873, *T*_max_ = 0.936
2572 measured reflections
2404 independent reflections
1420 reflections with *I* > 2σ(*I*)
*R*_int_ = 0.007
3 standard reflections every 180 reflections intensity decay: 1.2%

#### Refinement

*R*[*F* ^2^ > 2σ(*F* ^2^)] = 0.047
*wR*(*F* ^2^) = 0.150
*S* = 1.08
2404 reflections
178 parameters
H atoms treated by a mixture of independent and constrained refinement
Δρ_max_ = 0.21 e Å^−3^
Δρ_min_ = −0.28 e Å^−3^

### 

Data collection: *DIFRAC* (Gabe & White, 1993 ▶); cell refinement: *DIFRAC*; data reduction: *NRCVAX* (Gabe *et al.*, 1989 ▶); program(s) used to solve structure: *SHELXS97* (Sheldrick, 2008 ▶); program(s) used to refine structure: *SHELXL97* (Sheldrick, 2008 ▶); molecular graphics: *ORTEP-3* (Farrugia, 1997 ▶); software used to prepare material for publication: *SHELXL97* and *PLATON* (Spek, 2009 ▶).

## Supplementary Material

Crystal structure: contains datablocks I, global. DOI: 10.1107/S1600536809023897/su2117sup1.cif

Structure factors: contains datablocks I. DOI: 10.1107/S1600536809023897/su2117Isup2.hkl

Additional supplementary materials:  crystallographic information; 3D view; checkCIF report

## Figures and Tables

**Table 1:** Hydrogen-bond geometry (Å, °)

*D*—H⋯*A*	*D*—H	H⋯*A*	*D*⋯*A*	*D*—H⋯*A*
N1—H1*N*⋯O4	0.90 (4)	2.10 (4)	2.753 (3)	129 (3)
C13—H13⋯O3^i^	0.93	2.53	3.384 (4)	153

Symmetry code: (i) 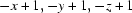.

**Fig. 1.**
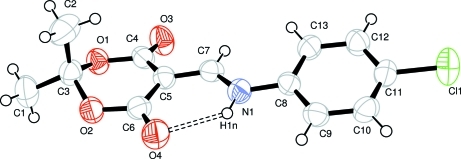
The molecular structure of the title compound, with displacement ellipsoids drawn at the 30% probability level.

**Fig. 2.**
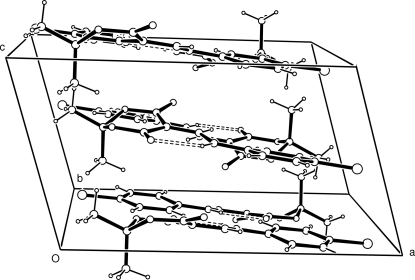
A crystal packing diagram of the title compound, showing the layer-like aggregation of the molecules in the unit cell.

## supplementary crystallographic information

### Comment

Quinolin-4-ol is an important model compound in the field of medicinal chemistry, and the synthesis of related compounds has been described previously (Cassis *et al.*, 1985). These compounds have been used as precursors for antitumor agents (Ruchelman *et al.*, 2003). 2,2-dimethyl-5- ((phenylamino)methylene)-1,3-dioxane-4,6-diones are the key intermediates to synthesize the quinolin-4-ol derivatives by thermal cracking (De *et al.*, 1998). The crystal structure of one such precursor, 5-(Aminomethylene)-2,2-dimethyl-1,3-dioxane-4,6-dione, has been descibed previously (de Silva *et al.*, 2006).

The title compound (Fig. 1) is approximately planar with a dihedral angle of 8.23 (4)° between the connecting aminomethylene unit and the planar part of the dioxane ring. Apart from that, the dioxane ring of the title compound exhibits a half-boat conformation, in which the C atom (C39) between the dioxane O-atoms is -0.464 (8) Å out-of-plane of the other five atoms. The molecule has an intramolecular N—H···O hydrogen bond which can stabilize the planar conformation (Table 1).

In the crystal the molecules stack in layers along the [001] direction (Fig. 2).

### Experimental

4-chlorobenzenamine(10 g,79.4 mmol), 2,2-dimethyl-1,3-dioxane-4,6-dione(13.6 g,94.1 mmol) and triethoxymethane(14 g,94.1 mmol) were suspended in ethanol at 363 K for 30 min. The white precipitate that formed was filtered off and recrystallized from acetone, giving colourless block-like crystals, suitable for X-ray diffraction analysis.

### Refinement

The NH H-atoms was located in a difference electron-density map and free refined: N-H = 0.90 (4) Å. The remainder of the H atoms were positioned geometrically (C—H = 0.93–0.96 Å) and refined using a riding model, with *U*_ĩso_(H) = 1.2 or 1.5*U*_eq_(C).

### Crystal data

**Table:**

C_13_H_12_ClNO_4_	*F*(000) = 584
*M**_r_* = 281.69	*D*_x_ = 1.437 Mg m^−^^3^
Monoclinic, *P*2_1_/*c*	Mo *K*α radiation, λ = 0.71073 Å
*a* = 13.439 (4) Å	Cell parameters from 24 reflections
*b* = 13.076 (3) Å	θ = 4.7–7.1°
*c* = 7.723 (3) Å	µ = 0.30 mm^−^^1^
β = 106.40 (2)°	*T* = 293 K
*V* = 1302.0 (7) Å^3^	Block, colourless
*Z* = 4	0.46 × 0.44 × 0.22 mm

### Data collection

**Table:**

Enraf–Nonius CAD-4 diffractometer	1420 reflections with *I* > 2σ(*I*)
Radiation source: fine-focus sealed tube	*R*_int_ = 0.007
graphite	θ_max_ = 25.5°, θ_min_ = 1.6°
ω/2θ scans	*h* = −9→16
Absorption correction: for a sphere (PROGRAM? REFERENCE?)	*k* = −15→0
*T*_min_ = 0.873, *T*_max_ = 0.936	*l* = −9→8
2572 measured reflections	3 standard reflections every 180 reflections
2404 independent reflections	intensity decay: 1.2%

### Refinement

**Table:**

Refinement on *F*^2^	Primary atom site location: structure-invariant direct methods
Least-squares matrix: full	Secondary atom site location: difference Fourier map
*R*[*F*^2^ > 2σ(*F*^2^)] = 0.047	Hydrogen site location: mixed
*wR*(*F*^2^) = 0.150	H atoms treated by a mixture of independent and constrained refinement
*S* = 1.08	*w* = 1/[σ^2^(*F*_o_^2^) + (0.0786*P*)^2^ + 0.0858*P*] where *P* = (*F*_o_^2^ + 2*F*_c_^2^)/3
2404 reflections	(Δ/σ)_max_ < 0.001
178 parameters	Δρ_max_ = 0.21 e Å^−^^3^
0 restraints	Δρ_min_ = −0.28 e Å^−^^3^

### Special details

**Table:**

Geometry. All e.s.d.'s (except the e.s.d. in the dihedral angle between two l.s. planes) are estimated using the full covariance matrix. The cell e.s.d.'s are taken into account individually in the estimation of e.s.d.'s in distances, angles and torsion angles; correlations between e.s.d.'s in cell parameters are only used when they are defined by crystal symmetry. An approximate (isotropic) treatment of cell e.s.d.'s is used for estimating e.s.d.'s involving l.s. planes.
Refinement. Refinement of *F*^2^ against ALL reflections. The weighted *R*-factor *wR* and goodness of fit *S* are based on *F*^2^, conventional *R*-factors *R* are based on *F*, with *F* set to zero for negative *F*^2^. The threshold expression of *F*^2^ > σ(*F*^2^) is used only for calculating *R*-factors(gt) *etc*. and is not relevant to the choice of reflections for refinement. *R*-factors based on *F*^2^ are statistically about twice as large as those based on *F*, and *R*- factors based on ALL data will be even larger.

### Fractional atomic coordinates and isotropic or equivalent isotropic displacement parameters (Å^2^)

**Table:**

	*x*	*y*	*z*	*U*_iso_*/*U*_eq_	
Cl1	0.05313 (6)	0.85662 (7)	0.57925 (14)	0.0831 (4)	
O1	0.71758 (14)	0.55344 (14)	0.3484 (3)	0.0558 (5)	
O2	0.76628 (14)	0.72663 (14)	0.4111 (3)	0.0583 (5)	
O3	0.55736 (15)	0.50190 (16)	0.3128 (3)	0.0713 (7)	
O4	0.65479 (16)	0.84296 (15)	0.4491 (3)	0.0697 (7)	
N1	0.46550 (18)	0.77642 (19)	0.4765 (3)	0.0515 (6)	
H1N	0.505 (3)	0.833 (3)	0.478 (5)	0.082 (11)*	
C1	0.8833 (2)	0.6088 (3)	0.3472 (5)	0.0759 (10)	
H1A	0.9068	0.5391	0.3581	0.114*	
H1B	0.9402	0.6532	0.4030	0.114*	
H1C	0.8566	0.6262	0.2219	0.114*	
C2	0.8321 (3)	0.5976 (3)	0.6385 (5)	0.0845 (11)	
H2A	0.7740	0.6062	0.6865	0.127*	
H2B	0.8868	0.6433	0.6990	0.127*	
H2C	0.8563	0.5283	0.6568	0.127*	
C3	0.7991 (2)	0.6212 (2)	0.4392 (4)	0.0554 (8)	
C4	0.6196 (2)	0.5702 (2)	0.3595 (4)	0.0520 (7)	
C5	0.5988 (2)	0.6710 (2)	0.4166 (4)	0.0474 (7)	
C6	0.6714 (2)	0.7528 (2)	0.4304 (4)	0.0510 (7)	
C7	0.5018 (2)	0.6873 (2)	0.4428 (4)	0.0502 (7)	
H7	0.4591	0.6306	0.4359	0.060*	
C8	0.3672 (2)	0.7933 (2)	0.5068 (4)	0.0458 (6)	
C9	0.3339 (2)	0.8928 (2)	0.5081 (4)	0.0552 (7)	
H9	0.3763	0.9466	0.4943	0.066*	
C10	0.2368 (2)	0.9123 (2)	0.5301 (4)	0.0598 (8)	
H10	0.2133	0.9792	0.5300	0.072*	
C11	0.1759 (2)	0.8326 (2)	0.5519 (4)	0.0539 (7)	
C12	0.2097 (2)	0.7335 (2)	0.5563 (4)	0.0579 (8)	
H12	0.1679	0.6801	0.5741	0.069*	
C13	0.3064 (2)	0.7134 (2)	0.5341 (4)	0.0552 (8)	
H13	0.3303	0.6465	0.5376	0.066*	

### Atomic displacement parameters (Å^2^)

**Table:**

	*U*^11^	*U*^22^	*U*^33^	*U*^12^	*U*^13^	*U*^23^
Cl1	0.0530 (5)	0.0953 (7)	0.1049 (8)	0.0101 (4)	0.0289 (5)	−0.0146 (5)
O1	0.0536 (11)	0.0520 (11)	0.0675 (13)	0.0026 (9)	0.0264 (10)	−0.0050 (10)
O2	0.0521 (11)	0.0511 (12)	0.0757 (14)	0.0003 (9)	0.0247 (10)	0.0013 (10)
O3	0.0645 (13)	0.0511 (12)	0.1081 (19)	−0.0080 (11)	0.0402 (13)	−0.0106 (12)
O4	0.0659 (13)	0.0443 (12)	0.1022 (19)	0.0001 (10)	0.0290 (13)	−0.0039 (11)
N1	0.0516 (13)	0.0486 (14)	0.0561 (16)	0.0029 (12)	0.0180 (12)	−0.0002 (11)
C1	0.0557 (18)	0.077 (2)	0.101 (3)	0.0016 (16)	0.0322 (18)	−0.008 (2)
C2	0.089 (2)	0.093 (3)	0.064 (2)	0.025 (2)	0.0100 (19)	0.0038 (19)
C3	0.0518 (16)	0.0520 (17)	0.063 (2)	0.0043 (14)	0.0175 (14)	−0.0017 (15)
C4	0.0551 (16)	0.0477 (16)	0.0598 (19)	0.0009 (14)	0.0270 (14)	0.0041 (14)
C5	0.0488 (14)	0.0464 (15)	0.0498 (17)	0.0015 (12)	0.0185 (13)	0.0004 (13)
C6	0.0554 (17)	0.0478 (17)	0.0504 (18)	0.0038 (13)	0.0159 (13)	0.0030 (13)
C7	0.0597 (17)	0.0468 (16)	0.0466 (17)	0.0020 (13)	0.0191 (14)	0.0037 (13)
C8	0.0471 (14)	0.0503 (16)	0.0413 (16)	0.0015 (12)	0.0147 (12)	0.0004 (13)
C9	0.0622 (17)	0.0482 (17)	0.0599 (19)	0.0001 (14)	0.0248 (15)	0.0039 (14)
C10	0.0685 (19)	0.0480 (17)	0.065 (2)	0.0133 (15)	0.0227 (16)	0.0032 (14)
C11	0.0458 (15)	0.0615 (18)	0.0534 (19)	0.0071 (14)	0.0122 (13)	−0.0020 (14)
C12	0.0530 (16)	0.0529 (18)	0.070 (2)	−0.0026 (14)	0.0221 (15)	−0.0012 (15)
C13	0.0579 (17)	0.0430 (15)	0.067 (2)	0.0071 (13)	0.0223 (15)	0.0016 (14)

### Geometric parameters (Å, °)

**Table:**

Cl1—C11	1.751 (3)	C2—H2B	0.9600
O1—C4	1.361 (3)	C2—H2C	0.9600
O1—C3	1.429 (3)	C4—C5	1.442 (4)
O2—C6	1.369 (3)	C5—C7	1.390 (4)
O2—C3	1.445 (3)	C5—C6	1.431 (4)
O3—C4	1.207 (3)	C7—H7	0.9300
O4—C6	1.216 (3)	C8—C9	1.377 (4)
N1—C7	1.318 (3)	C8—C13	1.378 (4)
N1—C8	1.422 (3)	C9—C10	1.387 (4)
N1—H1N	0.90 (4)	C9—H9	0.9300
C1—C3	1.505 (4)	C10—C11	1.364 (4)
C1—H1A	0.9600	C10—H10	0.9300
C1—H1B	0.9600	C11—C12	1.371 (4)
C1—H1C	0.9600	C12—C13	1.383 (4)
C2—C3	1.509 (5)	C12—H12	0.9300
C2—H2A	0.9600	C13—H13	0.9300
			
C4—O1—C3	119.4 (2)	C7—C5—C4	117.0 (2)
C6—O2—C3	118.4 (2)	C6—C5—C4	121.3 (2)
C7—N1—C8	125.7 (3)	O4—C6—O2	117.6 (3)
C7—N1—H1N	118 (2)	O4—C6—C5	126.1 (3)
C8—N1—H1N	116 (2)	O2—C6—C5	116.2 (2)
C3—C1—H1A	109.5	N1—C7—C5	125.4 (3)
C3—C1—H1B	109.5	N1—C7—H7	117.3
H1A—C1—H1B	109.5	C5—C7—H7	117.3
C3—C1—H1C	109.5	C9—C8—C13	120.5 (2)
H1A—C1—H1C	109.5	C9—C8—N1	117.8 (2)
H1B—C1—H1C	109.5	C13—C8—N1	121.7 (3)
C3—C2—H2A	109.5	C8—C9—C10	119.6 (3)
C3—C2—H2B	109.5	C8—C9—H9	120.2
H2A—C2—H2B	109.5	C10—C9—H9	120.2
C3—C2—H2C	109.5	C11—C10—C9	119.5 (3)
H2A—C2—H2C	109.5	C11—C10—H10	120.2
H2B—C2—H2C	109.5	C9—C10—H10	120.2
O1—C3—O2	111.0 (2)	C10—C11—C12	121.3 (3)
O1—C3—C1	106.0 (2)	C10—C11—Cl1	119.8 (2)
O2—C3—C1	105.7 (2)	C12—C11—Cl1	118.9 (2)
O1—C3—C2	109.7 (3)	C11—C12—C13	119.4 (3)
O2—C3—C2	109.7 (2)	C11—C12—H12	120.3
C1—C3—C2	114.6 (3)	C13—C12—H12	120.3
O3—C4—O1	117.6 (2)	C8—C13—C12	119.6 (3)
O3—C4—C5	126.2 (2)	C8—C13—H13	120.2
O1—C4—C5	116.1 (2)	C12—C13—H13	120.2
C7—C5—C6	121.4 (2)		
			
C4—O1—C3—O2	−44.6 (3)	C4—C5—C6—O2	−8.8 (4)
C4—O1—C3—C1	−158.9 (2)	C8—N1—C7—C5	−178.7 (3)
C4—O1—C3—C2	76.9 (3)	C6—C5—C7—N1	2.0 (5)
C6—O2—C3—O1	45.9 (3)	C4—C5—C7—N1	−172.6 (3)
C6—O2—C3—C1	160.4 (2)	C7—N1—C8—C9	−168.6 (3)
C6—O2—C3—C2	−75.6 (3)	C7—N1—C8—C13	11.4 (4)
C3—O1—C4—O3	−165.7 (3)	C13—C8—C9—C10	−2.5 (4)
C3—O1—C4—C5	17.8 (4)	N1—C8—C9—C10	177.5 (3)
O3—C4—C5—C7	8.9 (4)	C8—C9—C10—C11	0.6 (4)
O1—C4—C5—C7	−175.0 (2)	C9—C10—C11—C12	1.4 (5)
O3—C4—C5—C6	−165.8 (3)	C9—C10—C11—Cl1	−179.9 (2)
O1—C4—C5—C6	10.3 (4)	C10—C11—C12—C13	−1.5 (5)
C3—O2—C6—O4	162.9 (3)	Cl1—C11—C12—C13	179.7 (2)
C3—O2—C6—C5	−20.3 (4)	C9—C8—C13—C12	2.4 (4)
C7—C5—C6—O4	−6.8 (5)	N1—C8—C13—C12	−177.6 (3)
C4—C5—C6—O4	167.6 (3)	C11—C12—C13—C8	−0.4 (5)
C7—C5—C6—O2	176.8 (2)		

### Hydrogen-bond geometry (Å, °)

**Table:**

*D*—H···*A*	*D*—H	H···*A*	*D*···*A*	*D*—H···*A*
N1—H1N···O4	0.90 (4)	2.10 (4)	2.753 (3)	129 (3)
C13—H13···O3^i^	0.93	2.53	3.384 (4)	153

Symmetry codes: (i) −*x*+1, −*y*+1, −*z*+1.
